# Acute arterial mesenteric ischaemia: comparison of partial and complete occlusion of the superior mesenteric artery

**DOI:** 10.1186/s13244-025-01986-8

**Published:** 2025-05-08

**Authors:** Raphael Dufay, Lorenzo Garzelli, Iannis Ben Abdallah, Arnaud Tual, Dominique Cazals-Hatem, Olivier Corcos, Valérie Vilgrain, Emmanuel Weiss, Alexandre Nuzzo, Maxime Ronot

**Affiliations:** 1 Université Paris Cité, France & Service de Radiologie, Hôpital Beaujon, APHP.Nord, Clichy, France; 2 Service d’Imagerie Médicale et Interventionnelle, Hôpital Edouard Herriot, Hospices Civils de Lyon, Lyon, France; 3 Université Paris Cité, France & Service de Chirurgie Vasculaire, Hôpital Bichat, APHP.Nord, Paris, France; 4 Université Paris Cité, France & Service d’anatomopathologie, Hôpital Beaujon, APHP.Nord, Clichy, France; 5 Intestinal Stroke Center, Service de Gastroenterologie, MICI et Insuffisance Intestinale, Hôpital Beaujon, APHP.Nord, Clichy, France; 6 Université Paris Cité, France & Service d’anasthésie et de Réanimation, Hôpital Beaujon, APHP.Nord, Paris, France

**Keywords:** Mesenteric ischaemia, Mesenteric artery, Superior, Survival rate, Necrosis

## Abstract

**Objectives:**

To describe the characteristics and outcomes of patients with an incomplete occlusion of the superior mesenteric artery (SMA) (persistence of contrast-enhanced vessel lumen) and compare them to those with a complete occlusion of the SMA (complete interruption of the contrast-enhanced vessel lumen) in arterial acute mesenteric ischaemia (AMI).

**Material and methods:**

Retrospective study of arterial AMI patients (2006–2022). Demographics, laboratory tests, clinical characteristics, CT, treatments and outcomes were compared between patients with complete or incomplete SMA obstruction after adjusting for aetiology (embolic or atherosclerotic). The primary outcome was 30-day mortality, and the secondary outcome was 6-month gastrointestinal disability-free survival (no short bowel syndrome or parenteral nutritional support or permanent stoma).

**Results:**

151 patients (65 women, mean age 69) were included, 62 (41%) with incomplete and 89 (59%) with occlusive SMA occlusion. After adjusting for aetiology, chronic kidney failure (*p* = 0.03) and normal bowel enhancement on CT (*p* < 0.01) were associated with incomplete SMA occlusion. Patients with incomplete SMA occlusion were more frequently treated by endovascular revascularisation (*p* < 0.01) and stenting (*p* < 0.01), while patients with complete SMA occlusion were treated by open revascularisation. The 30-day mortality rate was 13% with no difference between incomplete (11%) and complete SMA occlusion (15%; *p* = 0.89). Nevertheless, complete SMA occlusion patients had a lower 6-month gastrointestinal disability-free survival rate (*p* = 0.01), more transmural necrosis (*p* < 0.01) and a higher risk of gastrointestinal disability (*p* = 0.02).

**Conclusion:**

Incomplete SMA occlusion can cause AMI with a similar 30-day mortality rate to completely occlusive forms. However, it is associated with poorer gastrointestinal outcomes, regardless of aetiology.

**Critical relevance statement:**

Acute arterial mesenteric ischaemia caused by incomplete occlusion of the superior mesenteric artery demonstrates similar 30-day mortality to complete occlusion but distinctively better gastrointestinal outcomes, emphasising nuanced imaging evaluation for targeted management strategies in these patients.

**Key Points:**

- Occlusive acute mesenteric ischaemia can be caused by incomplete superior mesenteric artery (SMA) occlusion.
- Acute mesenteric ischaemia caused by incomplete SMA occlusion has a similar 30-day mortality rate to complete SMA occlusion.
- A complete occlusion of the SMA is associated with poorer gastrointestinal outcomes

**Graphical Abstract:**

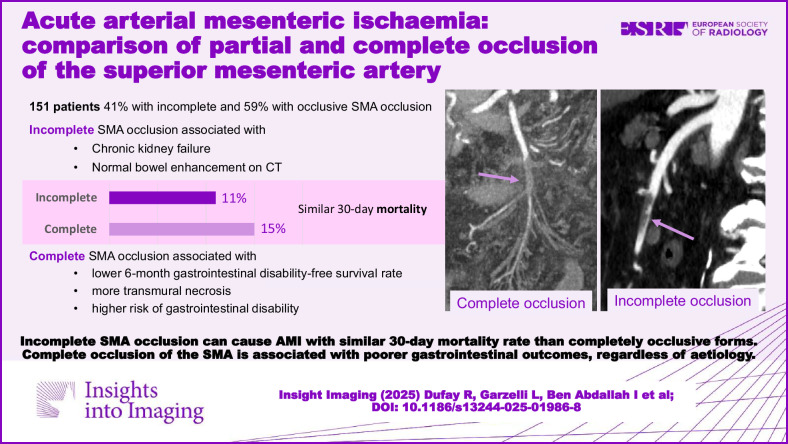

## Introduction

Arterial acute mesenteric ischaemia (AMI) is classically divided into “occlusive” and “non-occlusive” forms. Non-occlusive mesenteric ischaemia (NOMI) refers to a prolonged low-flow state in intensive care unit patients with splanchnic arteries that are patent but spasmed. Occlusive arterial acute mesenteric ischaemia is related to a lesion (usually atherosclerotic or embolic) of the superior mesenteric artery (SMA), causing a significant decrease in mesenteric blood flow and resulting in ischaemic bowel injuries. In these cases, it is generally thought that complete occlusion of the SMA lumen is needed to cause ischaemia. However, a clinically relevant incomplete SMA occlusion—without complete occlusion — may lead to AMI in a subset of patients (regardless of coeliac or inferior mesenteric arteries lesions). Although this entity is recognised and well-described in other end-organ ischaemia (e.g., intracranial or coronary diseases) [1, 2], it has not been clearly described or compared to actual complete SMA occlusion, and is not included in the guidelines for this disease [3, 4]. Whether an incomplete SMA occlusion has different clinical, biological and imaging presentations and outcomes than a complete SMA occlusion is largely unknown.

Thus, the aim of the study was to compare the outcomes and characteristics of patients with occlusive AMI according to degree of SMA occlusion (incomplete or complete).

## Material and methods

### Patient population

This IRB-approved single-centre retrospective observational study (CRM-2303-332) was performed according to the ethical standards of our institution’s Committee on Human Experimentation and reported according to the Strengthening the Reporting of Observational Studies in Epidemiology (STROBE) guidelines.

Patients with mesenteric ischaemia prospectively admitted to the mesenteric stroke centre unit of Beaujon Hospital (Clichy, France) were considered. Patients with arterial occlusive AMI and available pre-treatment computed tomography angiography (CTA) were included. Patients were excluded (1) if the cause of AMI was not embolic or atherosclerotic (e.g., dissection, vasculitis); (2) in case of in-stent or by-pass stenosis/occlusion in patients with a history of revascularised SMA; (3) in the presence of a celiacomesenteric trunk; (4) with a history of bowel resection or 5) with isolated coeliac trunk occlusion and upper gastrointestinal ischaemic injury only.

Demographics, medical history, clinical characteristics, laboratory tests (serum lactate, white blood cell (WBC), creatinine, C-reactive protein (CRP)), aetiology (embolic or atherosclerotic), and date of the onset of symptoms were recorded.

### Diagnosis of acute mesenteric ischaemia

The diagnosis of AMI was based on an association of (1) acute intestinal symptoms (e.g., intense and sudden pain, mostly requiring opioids, diarrhoea), (2) arterial involvement on CTA (including stenosis or occlusion of the SMA), (3) related bowel abnormalities consistent with ischaemic injury on CT (e.g., decreased bowel wall enhancement, bowel wall pneumatosis, bowel wall thickening [5]), and (4) the absence of a competing cause. Bowel abnormalities could be missing in early forms of the disease, and in these cases, a diagnosis of AMI was considered if the three other criteria were met. The final diagnosis was determined by an on-call group of AMI experts, including gastroenterologists, surgeons, and radiologists.

AMI patients were divided into two groups based on SMA anomalies, which were retrospectively classified as complete or incomplete occlusion (Supplemental Figures). Because there are no guidelines on how to grade or measure an incomplete SMA occlusion, the recommendations from the Society of Cardiovascular Computed Tomography were applied [6]. Obstructive embolic or atherosclerotic lesions were evaluated with multi-planar reformation imaging using an orthogonal plane perpendicular to the SMA axis. A complete SMA occlusion was defined as complete occlusion of the SMA with complete interruption of the contrast-enhanced vessel lumen. Otherwise, the image was defined as incomplete SMA occlusion. Patients with concomitant occlusion and stenosis of the SMA were classified as complete SMA occlusion. Both forms could be from embolic or atherosclerotic aetiology.

The SMA was retrospectively segmented according to Tual et al as proximal (S1, between the ostium and the origin of the inferior pancreaticoduodenal artery), middle (S2, between the origin of the first jejunal artery and the origin of the ileocolic artery), and distal (S3, after the origin of the ileocolic artery) [7]. The aetiology was considered to be an arterial embolism when other infarcts were observed on CTA or in case of a hypodense clot with no underlying atherosclerosis of the SMA. SMA, the coeliac trunk, and the inferior mesenteric or abdominal aorta circumferential calcifications were reported. The largest diameter of the origin of the inferior mesenteric and gastroduodenal arteries was measured. The key CT features of ischaemic bowel injury were also recorded: decreased bowel enhancement, bowel dilatation (25 mm) and bowel wall pneumatosis [8, 9]. Any colon involvement was also noted [10]. No angiography was performed for diagnostic purposes. All CTs were reviewed in consensus by two radiologists (R.D. and L.G.) with 4 and 6 years of experience in reviewing AMI features (vascular and digestive). CT parameters (for the CT performed in our centre) are provided in the Supplementary Table 1.

### Treatment strategy

All admitted patients were treated according to the institutional protocol established by Corcos et al and described in previous studies [11–13]. It is provided in details as Supplemental material.

### Endpoints

The primary outcome was the 30-day mortality rate, as frequently reported in AMI [14–21]. The secondary outcomes were the rate of gastrointestinal disability-free survival and the 3-month, 6-month, and 12-month overall survival rates. The gastrointestinal disability-free survival was defined as the absence of short bowel syndrome, parenteral nutrition, or a definite stoma in 30-day survivors and was assessed at 3-, 6-, and 12-months.

### Sample size and statistical analysis

We expected a complete/incomplete SMA occlusion ratio of 2/1. The primary outcome (i.e., 30-day mortality) was estimated to be around 50% in patients with a complete SMA occlusion based on the most recent meta-analysis [22] and around 20% in an incomplete SMA occlusion based on estimations in the most comparable populations. Thirty-day mortality was 16% in a study evaluating acute-on-chronic AMI (with 47% stenotic-only patients) [23] and 28% in a study of atherosclerotic AMI (with 25% stenotic-only patients) [24]. At least 84 patients were needed for the sample size to meet the primary outcome (30-day mortality) with a statistical power of 80%.

Results are presented as means ± standard deviations (SD) or the number of cases (percentage of cases). Patients with incomplete and complete SMA occlusion were compared with the Student *t*-test for normally distributed continuous variables (the Mann–Whitney test was used for non-normal variables) and the Chi-square test for categorical variables. Then, each variable was adjusted for aetiology (atherosclerotic vs. embolic) by logistic regression for continuous variables or by the Cochran-Mantel-Haenszel test for categorical variables. Adjustments on aetiology were systematically performed because we expected the results to be biased by the strong associations between embolic cause and a complete SMA occlusion, as well as between atherosclerotic cause and an incomplete SMA occlusion. Tests were two-sided, and *p* < 0.05 was considered to be significant. All analyses were performed using Stata version 14.0.

## Results

### Patient history, clinical, and laboratory tests

**Fig. 1:**
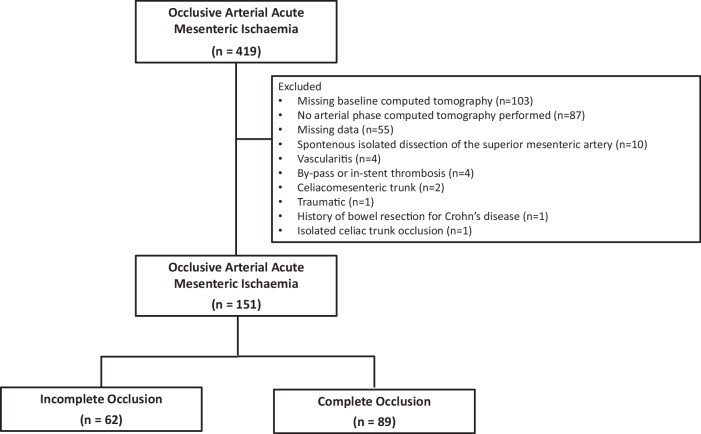
Study flow chart

Four hundred nineteen patients with AMI admitted to the mesenteric stroke unit were screened. After applying inclusion and exclusion criteria, 151 patients (65 women, 43%), mean age 69 years old (29–91) were included (Fig. 1). The patients’ characteristics are provided in Table 1. Overall, 89 (59%) patients had a complete SMA occlusion, and 62 (41%) had an incomplete SMA occlusion.

**Table 1:** Patient’s history, clinical, and laboratory characteristics without and with adjusting for aetiology (i.e., atherosclerotic vs. embolic)

	All patients *N* = 151	Incomplete occlusion *N* = 62	Complete occlusion *N* = 89	*p*		Adjusted *p*
Female	65 (43%)	20 (32%)	45 (51%)	0.02	0.25	
Age, mean (years)	69 (± 1)	69 (± 1)	69 (± 1)	0.86	0.69	
BMI, mean (kg.m^−2^)^33^	26.1 (± 5.6)	26.4 (± 5.7)	26.0 (± 5.5)	0.66	0.23	
Patient’s history						
Tobacco use^3^	80 (54%)	37 (61%)	43 (50%)	0.24	0.80	
Arterial hypertension	108 (72%)	50 (81%)	68 (65%)	0.03	0.06	
Dyslipidaemia	58 (38%)	26 (42%)	32 (36%)	0.45	0.99	
Diabetes mellitus	56 (37%)	27 (44%)	29 (33%)	0.17	0.16	
Myocardial infarction	31 (21%)	17 (27%)	15 (17%)	0.11	0.11	
Limb ischaemia	38 (25%)	21 (34%)	17 (19%)	0.04	0.53	
Atrial fibrillation^1^	51 (34%)	18 (30%)	33 (37%)	0.33	0.18	
Chronic kidney disease^1^	22 (15%)	14 (23%)	8 (9%)	0.01	**0.03**	
Symptoms						
Initial abdominal pain^1^	146 (97%)	58 (95%)	88 (99%)	0.15	0.47	
Need for opioid^8^	14 (10%)	7 (12%)	7 (8%)	0.44	0.76	
Persistent abdominal pain^9^	85 (60%)	30 (52%)	55 (65%)	0.10	0.21	
Initial diarrhoea^1^	58 (39%)	24 (39%)	34 (38%)	0.88	0.84	
Bloody diarrhoea^1^	29 (19%)	13 (21%)	16 (18%)	0.61	0.88	
Biological examination						
Initial lactate (mmol/L), mean^11^	2.4 (± 2.1)	1.9 (± 1.0)	2.8 (± 2.5)	0.01	0.09	
Lactate > 2 mmol/L	58 (41%)	18 (32%)	40 (48%)	0.05	0.11	
Initial CRP (mg/L), mean^37^	121 (± 113)	131 (± 118)	112 (± 109)	0.38	0.91	
Initial WBC count (G/L), mean^12^	16.0 (± 6.9)	14.5 (± 6.9)	17.0 (± 6.7)	0.03	0.06	
Initial creatinine (µmol/L), mean^19^	128 (± 134)	148 (± 154)	114 (± 116)	0.14	0.31	
Delay symptoms—CT, days^6^	4.8 (± 22)	6.8 (± 35)	3.5 (± 8)	0.39	0.67	
Embolic cause	64 (42%)	12 (19%)	52 (58%)	< 0.01		

Bold values correspond to signifiant statistical tests

Superscript numbers correspond to the number of missing data for the corresponding variable

*BMI* body mass index, *CRP* C-reactive protein, *CT* computed tomography, *WBC* white blood cells

There were more women in the complete occlusion group (45/89; 51% vs. 20/62; 32%; *p* = 0.02). Patients with a complete SMA occlusion had less arterial hypertension than those with an incomplete SMA occlusion (68/89 (65%) vs. 50/62 (81%); *p* = 0.03), less limb ischaemia (17/89 (19%) vs. 21/62 (34%); *p* = 0.04) and less chronic renal failure (8/89 (9%) vs. 14/61 in (23%); *p* = 0.01). Chronic renal failure was the only factor associated with an incomplete SMA occlusion after adjusting for aetiology (*p* = 0.03).

The aetiology of AMI was strongly associated with the type of AMI. A complete SMA occlusion was present in 52/64 (81%) and 37/87 (43%) patients with embolic and atherosclerotic AMI, respectively (*p* < 0.01).

Laboratory analyses showed that patients with a complete SMA occlusion had higher initial serum lactate (2.8 vs. 1.9 mmol/L in those with an incomplete SMA occlusion; *p* = 0.01) and a higher initial WBC (17.0 vs. 14.5 G/L in those with an incomplete SMA occlusion; *p* = 0.03). There were no significant differences between the complete and incomplete groups after adjusting for aetiology.

The delay between the onset of symptoms and the initial CT (diagnosis) was 4.8 days for the whole cohort, 6.8 days in the incomplete and 3.5 days in the complete SMA occlusion groups, without statistical difference between the two groups after adjusting for aetiology (*p* = 0.67).

### Computed tomography angiography features

CT features before treatment (i.e., revascularisation with or without bowel resection) are provided in Table 2.

**Table 2:** Computed tomography angiography features and Clichy score before treatment without and with adjusting for aetiology (atherosclerotic vs. embolic)

	All patients *N* = 151	Incomplete occlusion *N* = 62	Complete occlusion *N* = 89	*p*	Adjusted *p*
Vascular analysis					
Main SMA lesion^a^				< 0.01	0.27
S1 (proximal)	81 (54%)	45 (73%)	36 (40%)		
S2 (middle)	52 (34%)	14 (22%)	38 (43%)		
S3 (distal)	18 (12%)	3 (5%)	15 (17%)		
SMA calcification	56 (37%)	33 (53%)	23 (26%)	0.01	0.12
Coeliac trunk calcification	36 (24%)	23 (37%)	13 (15%)	< 0.01	0.18
Gastroduodenal artery diameter, mean (mm)	3.3 (± 0.1)	3.5 (± 0.2)	3.2 (± 0.1)	0.12	0.06
IMA stenosis grade				0.12	0.51
< 50%	108 (72%)	42 (68%)	66 (74%)		
> 50%	26 (17%)	15 (24%)	11 (12%)		
Occlusion	17 (11%)	5 (8%)	12 (13%)		
IMA calcification	19 (13%)	13 (21%)	6 (7%)	< 0.01	0.13
IMA diameter, mean (mm)	2.5 (± 0.1)	2.6 (± 0.2)	2.5 (± 0.1)	0.51	0.20
Abdominal aorta circumferential calcification	83 (65%)	41 (66%)	42 (47%)	0.02	0.57
Bowel analysis					
Decreased wall enhancement	64 (43%)	17 (27%)	47 (54%)	< 0.01	**<** **0.01**
Bowel dilatation (≥ 25 mm)	59 (39%)	21 (34%)	38 (43%)	0.25	0.20
Bowel wall pneumatosis	17 (11%)	10 (16%)	7 (8%)	0.12	0.35
Colon involvement	21 (14%)	13 (21%)	8 (9%)	0.03	0.29
Maximum Clichy Score [12]				0.36	0.10
0	45 (32%)	23 (40%)	22 (27%)		
1	51 (37%)	21 (36%)	30 (37%)		
2	30 (22%)	10 (17%)	20 (25%)		
3	13 (9%)	4 (7%)	9 (11%)		
2/3 (vs. 0/1)	43 (31%)	14 (24%)	29 (36%)	0.14	0.27

Bold values correspond to signifiant statistical tests

Superscript numbers correspond to the number of missing data for the corresponding variable

*IMA* inferior mesenteric artery, *SMA* superior mesenteric artery

^a^ Per Tual et al [7]

Patients with a complete SMA occlusion had more S2 (38/89, 43%) or S3 (15/89, 17%) lesions, while those with an incomplete SMA occlusion had more S1 (45/62, 73%; *p* < 0.01) lesions. The rate of calcifications, coeliac trunk calcifications, inferior mesenteric artery calcifications and abdominal aorta calcifications was higher in the incomplete group (33/62 (53%) vs. 23/89 (26%), *p* = 0.01), (23/62 (37%) vs. 13/89 (15%); *p* < 0.01), (13/62 (21%) vs. 6/89 (7%); *p* < 0.01), and (41/62 (66%) vs. 42/89 (47%); *p* = 0.02), respectively. The mean diameter of the gastroduodenal artery was greater in the incomplete group (3.5 vs. 3.2 mm, *p* = 0.02). These differences were not maintained after adjustment for aetiology.

Bowel analysis showed that decreased bowel enhancement was more frequent in complete (47/87, 54%) than in incomplete SMA occlusion (17/62, 27%; *p* < 0.01; two missing data due to absent portal venous phase images), and colonic involvement was more frequent in patients with an incomplete SMA occlusion (13/62 (21%) vs. 8/89, (9%); *p* = 0.03). After adjusting for aetiology, only decreased bowel wall enhancement was associated with a complete SMA occlusion (*p* < 0.01).

### Treatment and outcomes

Treatment characteristics and outcomes are provided in Table 3.

**Table 3:** Invasive treatments and outcomes without and with adjusting for aetiology (atherosclerotic vs. embolic)

	All patients *N* = 151	Incomplete occlusion *N* = 62	Complete occlusion *N* = 89	*p*	Adjusted *p*
Interval initial CT—revascularisation, mean days (SD)	3.6 (4.5)	2.3 (3.3)	1.9 (5.2)	0.63	0.52
Revascularisation					
Absent	18 (12%)	4 (6%)	14 (16%)	0.08	0.31
Endovascular	93 (62%)	50 (81%)	43 (48%)	< 0.01	**<** **0.01**
Stenting	56 (37%)	40 (65%)	16 (18%)	0.01	**<** **0.01**
Thrombolysis	33 (22%)	11 (18%)	22 (25%)	0.30	0.29
Thrombectomy	29 (19%)	7 (11%)	22 (25%)	0.03	0.26
Open	54 (36%)	11 (18%)	43 (48%)	< 0.01	**<** **0.01**
Reperfusion injury	21 (14%)	5 (8%)	16 (18%)	0.08	0.26
Bowel resection	80 (53%)	27 (44%)	53 (60%)	0.05	0.05
Transmural necrosis on pathology^19^	41 (31%)	10 (19%)	31 (40%)	0.01	**<** **0.01**
Long-term gastrointestinal disability	51 (34%)	15 (24%)	36 (40%)	0.03	**0.01**
Short bowel syndrome^1^	41 (27%)	12 (20%)	29 (33%)	0.08	0.07
Parenteral nutrition^7^	35 (24%)	11 (18%)	24 (29%)	0.15	0.20
Permanent stoma^5^	20 (14%)	7 (11%)	13 (15%)	0.49	0.17
Endpoints					
30-day mortality rate	13% (20/151)	11% (7/62)	15% (13/89)	0.54	0.89
All-cause mortality rate					
3-month	19% (28/151)	16% (10/62)	20% (18/89)	0.52	0.62
6-month	21% (32/151)	16% (10/62)	25% (22/89)	0.20	0.25
12-month	27% (41/151)	23% (14/62)	30% (27/89)	0.29	0.29
Gastrointestinal disability-free survival rate					
3-month	55% (83/151)	66% (41/62)	47% (42/89)	0.02	**0.01**
6-month	53% (80/151)	66% (41/62)	44% (39/89)	< 0.01	**<** **0.01**
12-month	51% (77/151)	61% (38/62)	44% (39/89)	0.03	**0.01**

Bold values correspond to signifiant statistical tests

Superscript numbers correspond to the number of missing data for the corresponding variable

*CT* computed tomography

The interval between initial CT and treatment was 3.6 days for the whole cohort, 2.3 days in an incomplete and 3.9 days in complete SMA occlusion without statistical difference between the two groups after adjusting for aetiology (*p* = 0.52).

Patients with an incomplete SMA occlusion were more frequently treated by endovascular revascularisation (50/62 (81%) vs. 43/89(48%); *p* < 0.01) and especially by stenting (40/62 (65%) vs. 16/89 (18%); *p* < 0.01) before and after adjusting for aetiology. On the other hand, patients with a complete SMA occlusion more frequently underwent open revascularisation (43/89, 48% vs. 11/62, 18%); *p* < 0.01, before and after adjusting for aetiology). Of note, 18 patients (4 incomplete and 14 complete) were not revascularised in the cohort. Patients with a complete SMA occlusion had higher rates of transmural necrosis on pathology (31/78, 40% vs. 10/54, 19%; *p* = 0.01 before and *p* < 0.01 after adjustment for aetiology).

The median follow-up was 30 months (IQR 12–75). The 30-day mortality rate was 20/151 (13%). All-cause mortality was 28/151 (19%) at 3 months, 32/151 (21%) at 6 months and 41/151 (27%) one year after AMI. Among the 131 30-day survivors, 83/131 (63%) were alive and gastrointestinal disability-free at 3-months, 80/131 (61%) at 6-months, and 77/131 (59%) at 12-months, resulting in 3-month, 6-month, and 12-month gastrointestinal disability-free survival rates of 55%, 53%, and 51%, respectively.

No difference was found in the 30-day mortality rates between the incomplete and complete groups before (*p* = 0.54) or after (*p* = 0.89) adjusting for aetiology and other confounding factors (i.e., sex, age, hypertension, clinical presentation, arterial calcifications and the diameter of the IMA).

**Fig. 2:**
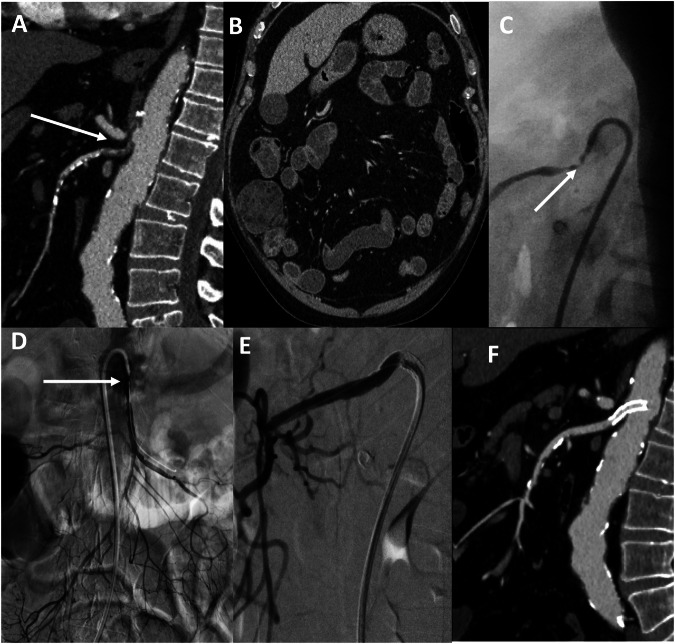
A seventy-six-year-old patient with chronic kidney disease and a history of chronic mesenteric ischaemia was admitted for acute mesenteric ischaemia. He presented a marked abdominal pain with initial diarrhoea and CRP increase (194 mg/L). On computed tomography angiography (CTA), an incomplete SMA occlusion (lumen stenosis > 90%) of the superior mesenteric artery (SMA) was depicted (**A**, arrow) with normal bowel enhancement (**B**). Digital non-subtracted and subtracted angiograms confirm the incomplete occlusion of the SMA (**C**, **D**, arrows). Completion subtracted angiogram (**E**) and CTA (**F**) after mesenteric artery stenting illustrate complete revascularisation without residual stenosis. This patient did not require bowel resection and recovered rapidly

**Fig. 3:**
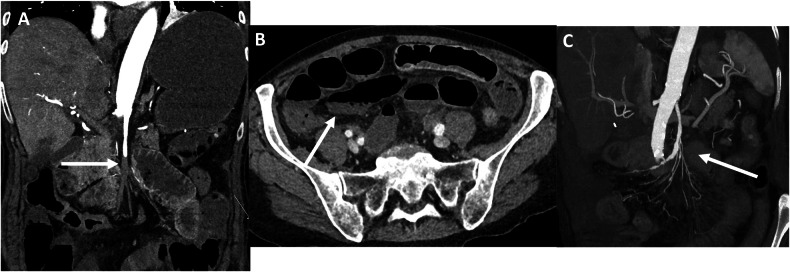
Seventy-eight-year-old patient with acute mesenteric ischaemia due to a complete occlusion of the superior mesenteric artery (embolic). He presented to the emergency department with marked acute abdominal pain and a hyperleukocytosis of 22.7 G/L. Occlusion of the middle SMA (S2 segment) (**A**, arrow) with a decreased enhancement of the bowel with dilatation (**B**, arrow). This patient was successfully treated by open thrombectomy with patch angioplasty, leading to a complete revascularisation of the SMA and its branches (**C**, arrow). This patient underwent subsequent bowel resection with only 50 cm of jejunum left in place, leading to a short bowel syndrome with a parenteral nutritional support dependence

However, among the 30-day survivors, patients with a complete SMA occlusion experienced more gastrointestinal disability (32/76, 42%) than those with an incomplete SMA occlusion (13/55, 24%); *p* = 0.02 before and *p* = 0.01 after adjustment for aetiology. Patients with a complete SMA occlusion also had lower 3-month, 6-month and 12-month gastrointestinal disability-free survival rates (*p* = 0.01, *p* < 0.01, and *p* = 0.01, respectively) (Figs. 2 and 3).

## Discussion

This study describes and compares the presentation and outcomes of patients with AMI due to an incomplete or complete SMA occlusion. Incomplete SMA occlusions represented 41% of the AMI cohort, which is an important percentage. We believe that the classic opposition between “occlusive” and “non-occlusive” AMI (NOMI) may be misleading. The pathophysiology of incomplete SMA occlusions is close to that of occlusive AMI because it is based on a local haemodynamic impairment of the bowel arterial support, as opposed to diffuse systemic anomalies in NOMI, where the small bowel is not the only organ to suffer from ischaemia.

After adjusting for aetiology, chronic kidney disease and the presence of bowel enhancement on pre-treatment CTA were more frequent in patients with an incomplete SMA occlusion. The association between chronic kidney disease and stenotic forms of AMI is consistent with the literature. Chronic kidney disease has been found to be associated with more severe stenosis of the SMA [25], increased mortality [19, 20, 26, 27] and re-admission after revascularisation [28] in patients with chronic or AMI.

Data on the association between bowel wall enhancement and the type of SMA lesion are scarce. Certain studies have evaluated the prognostic value of decreased bowel enhancement. For example, Canfora et al [29] reported that a decreased enhancement was significantly associated with transmural necrosis, but only on univariable analysis. Decreased enhancement on the initial CT has also been found to be associated with the development of reperfusion injury after endovascular revascularisation, suggesting a more advanced stage of ischaemia [13]. Since bowel enhancement is directly linked to its perfusion, one may hypothesise that stenotic forms occur in patients with pre-existing arterial remodelling triggered by progressive arterial stenosis and leading to the development of collaterals. However, the calibre of the inferior mesenteric artery was not different between groups, and not all stenotic forms were due to atherosclerosis. Moreover, a complete occlusion of the SMA (regardless of the cause) may have more impact on bowel perfusion and result in less bowel enhancement.

Patients with an incomplete SMA occlusion more frequently underwent endovascular revascularisation and stenting than those with a complete SMA occlusion, while the latter more frequently underwent open revascularisation. The location and pattern of SMA lesions mainly explain these findings. The incomplete SMA occlusion was more frequently due to proximal lesions (i.e., S1), which respond better to endovascular revascularisation. On the other hand, complete occlusion of the SMA was more often distal and ramified, associated with a higher risk of technical failure with endovascular approaches, thus favouring the open approach.

We decided to adjust comparisons for aetiology (i.e., embolic vs. atherosclerotic) since this is certainly a confounding factor. Clots usually lead to complete occlusion of the SMA (the clot fully blocks the vessel), while atherosclerotic lesions slowly progress from stenosis to occlusion, with plaque remodelling leading to instability. Moreover, those two aetiologies are related to entirely different mechanisms and are associated with different patient baseline characteristics (heart arrhythmia in embolic forms vs. diffuse atherosclerotic diseases in atherosclerotic SMA) and outcomes, with a supposedly better prognosis in embolic forms than in atherosclerotic ones [30]. For example, arterial hypertension, limb ischaemia, the location of the SMA lesions and vessel calcifications were all associated with atherosclerosis and thus significantly associated with an incomplete SMA occlusion before but not after adjusting for aetiology. Before adjustment for aetiology, one could argue that our study actually compared embolic and atherosclerotic forms of AMI, rather than stenotic and occlusive forms *per se*. However, because the aetiologies and forms of vascular lesions did not entirely overlap, we believe that providing both unadjusted and adjusted results was more clinically relevant.

The 30-day mortality rate in our cohort was 13%, with no difference between the complete and incomplete SMA occlusions. The 30-day mortality rate is the most frequently reported outcome in AMI [14–22]. We report one of the lowest 30-day mortality rates in AMI in the literature. For instance, in the recent meta-analysis by Tamme et al [22], the 30-day mortality rate was 51.8% (61 studies). It was 33.9% (24 studies) in revascularised patients. Our excellent results must be interpreted in relation to the setting of our multidisciplinary digestive vascular unit dedicated to mesenteric ischaemia, which we believe is a key determinant in patients' survival rate. Intervals between symptom onset and diagnosis, or between diagnosis and treatment, were not significantly different between the incomplete and complete SMA occlusion groups, even after adjusting for aetiology, illustrating that the vascular lesion is not sufficient to shorten the diagnosis or the treatment. Yet a longer interval between the diagnosis and revascularisation was found to increase the 30-day mortality rate [31] and this impact of time upon outcome should be better investigated.

With these 30-day mortality rates, the study was underpowered because obtaining a 5% difference between the incomplete and complete SMA occlusion groups would require over 1500 patients. Even a nationwide registry cohort cannot meet this objective [22, 32]. This emphasises that, as mortality rates of AMI decrease over time with appropriate multidisciplinary management [30, 32], the primary outcome of AMI studies should include composite criteria combining mortality and gastrointestinal outcomes defined as the absence of a major gastrointestinal negative event (i.e., short bowel syndrome, parenteral nutrition, or definite stoma) similar to the modified Rankin Scale in stroke trials [33]. In this perspective, patients with a complete SMA occlusion had a higher rate of transmural necrosis on pathology and permanent gastrointestinal disability as well as a lower rate of gastrointestinal disability-free survival, suggesting more severe ischaemic injury.

The first limitation of this study is its retrospective design. This explains why a subset of initially screened patients were excluded for missing data, mostly because baseline CTA were undertaken in primary or secondary care hospitals before being transferred to our dedicated unit and were not available for review anymore. Also, the differentiation between the incomplete and complete SMA occlusion groups may be challenging in heavily calcified lesions because of beam hardening artefacts along with a partial volume effect caused by the limited spatial resolution. These may have led to mislabelling of severe stenosis and complete occlusion. This issue is well-documented in coronary CT with several studies focusing on differentiating complete occlusion from severe stenosis [34]. However, it is unlikely that any possible mislabelling would have generated significant bias on presentation or outcomes. It is important to keep in mind that, unlike the North American Symptomatic Carotid Endarterectomy Trial (NASCET) or the Society of Coronary Computed Tomography (SCCT) for carotid or coronary arteries, respectively, there is no consensus to date on how to measure or grade an SMA stenosis. We did not evaluate the inter-reader agreement for the various AMI features, as it was performed in a previous study published by our group [35]. In this study, the inter-reader agreement for differentiating stenosis vs. occlusion of the SMA was substantial/good (kappa = 0.78; 88% of agreement). Finally, these results come from a European AMI stroke centre, which we consider a strength in terms of clinical treatment. However, it also represents a limitation when assessing external validity.

In conclusion, we showed that “occlusive AMI” could be caused by an incomplete occlusion of the SMA. Patients with an incomplete vascular occlusion had more frequent chronic kidney disease, persistent bowel enhancement on pre-treatment CT, and more frequently underwent endovascular revascularisation with stenting compared to patients with a complete SMA occlusion. Furthermore, while complete and incomplete occlusion forms of AMI had the same 30-day mortality rate, patients with completely occlusive forms had higher rates of transmural necrosis, long-term gastrointestinal disability and lower gastrointestinal disability-free survival. This suggests that complete occlusion of the SMA is associated with a worse prognosis, regardless of aetiology.

## Supplementary information

ELECTRONIC SUPPLEMENTARY MATERIAL

## Data Availability

Data and materials and be accessed upon reasonable request to the corresponding author.

## Abbreviations

AMI: Acute mesenteric ischaemia
CRP: C-reactive protein
CTA: Computed tomography angiography
NOMI: Non-occlusive mesenteric ischaemia
SMA: Superior mesenteric artery
WBC: White blood cell

## References

[1] White H, Boden-Albala B, Wang C et al (2005) Ischemic stroke subtype incidence among whites, blacks, and Hispanics: the Northern Manhattan Study. Circulation 111:1327–1331 15769776 10.1161/01.CIR.0000157736.19739.D0

[2] Ambrose JA, Tannenbaum MA, Alexopoulos D et al (1988) Angiographic progression of coronary artery disease and the development of myocardial infarction. J Am Coll Cardiol 12:56–62 3379219 10.1016/0735-1097(88)90356-7

[3] Bala M, Catena F, Kashuk J et al (2022) Acute mesenteric ischemia: updated guidelines of the World Society of Emergency Surgery. World J Emerg Surg 17:54 36261857 10.1186/s13017-022-00443-x PMC9580452

[4] Björck M, Koelemay M, Acosta S et al (2017) Editor’s Choice—Management of the diseases of mesenteric arteries and veins: clinical practice guidelines of the European Society of Vascular Surgery (ESVS). Eur J Vasc Endovasc Surg 53:460–510 28359440 10.1016/j.ejvs.2017.01.010

[5] Alpern MB, Glazer GM, Francis IR (1988) Ischemic or infarcted bowel: CT findings. Radiology 166:149–152 3336673 10.1148/radiology.166.1.3336673

[6] Raff GL, Abidov A, Achenbach S et al (2009) SCCT guidelines for the interpretation and reporting of coronary computed tomographic angiography. J Cardiovasc Comput Tomogr 3:122–136 19272853 10.1016/j.jcct.2009.01.001

[7] Tual A, Garzelli L, Nuzzo A et al (2023) Strengthening the description of superior mesenteric artery occlusions in acute mesenteric ischaemia: proposition for an anatomical classification. Eur J Vasc Endovasc Surg 65:802–808. 10.1016/j.ejvs.2023.01.041 36736617

[8] Nuzzo A, Maggiori L, Ronot M et al (2017) Predictive factors of intestinal necrosis in acute mesenteric ischemia: prospective study from an intestinal stroke center. Am J Gastroenterol 112:597–605 28266590 10.1038/ajg.2017.38

[9] Zeng Y, Yang F, Hu X, Zhu F, Chen W, Lin W (2022) Radiological predictive factors of transmural intestinal necrosis in acute mesenteric ischemia: systematic review and meta-analysis. Eur Radiol 33:2792–2799 10.1007/s00330-022-09258-5 36449058

[10] Ksouri A, Copin P, Bonvalet F et al (2022) Colonic involvement in acute mesenteric ischemia: prevalence, risk factors, and outcomes. Eur Radiol 32:2813–2823 34657969 10.1007/s00330-021-08318-6

[11] Corcos O, Castier Y, Sibert A et al (2013) Effects of a multimodal management strategy for acute mesenteric ischemia on survival and intestinal failure. Clin Gastroenterol Hepato 11:158–165.e2 10.1016/j.cgh.2012.10.027 23103820

[12] Nuzzo A, Maggiori L, Paugam-Burtz C et al (2019) Oral antibiotics reduce intestinal necrosis in acute mesenteric ischemia: a prospective cohort study. Am J Gastroenterol 114:348–351 30538292 10.1038/s41395-018-0389-9

[13] Garzelli L, Nuzzo A, Hamon A et al (2022) Reperfusion injury on computed tomography following endovascular revascularization of acute mesenteric ischemia: prevalence, risk factors, and patient outcome. Insights Imaging 13:194 36512135 10.1186/s13244-022-01339-9 PMC9748024

[14] Block TA, Acosta S, Björck M (2010) Endovascular and open surgery for acute occlusion of the superior mesenteric artery. J Vasc Surg 52:959–966 20620006 10.1016/j.jvs.2010.05.084

[15] Garzelli L, Abdallah IB, Nuzzo A, Corcos O, Castier Y, Ronot M (2022) Endovascular thrombectomy for acute arterial mesenteric ischaemia: no benefit of mechanical over manual thrombus aspiration. Eur J Vasc Endovasc Surg 64:128–129 10.1016/j.ejvs.2022.05.020 35568314

[16] Sénémaud JN, Roussel A, Pellenc Q et al (2021) Retrograde open mesenteric stenting for acute and chronic mesenteric ischaemia: results from an intestinal stroke centre. Eur J Vasc Endovasc Surg 62:55–63 33965329 10.1016/j.ejvs.2021.03.019

[17] Pedersoli F, Schönau K, Schulze-Hagen M et al (2021) Endovascular revascularization with stent implantation in patients with acute mesenteric ischemia due to acute arterial thrombosis: clinical outcome and predictive factors. Cardiovasc Interv Radio 44:1030–1038 10.1007/s00270-021-02824-2 PMC8190006 33825061

[18] Jia Z, Jiang G, Tian F et al (2014) Early endovascular treatment of superior mesenteric occlusion secondary to thromboemboli. Eur J Vasc Endovasc Surg 47:196–203 24183620 10.1016/j.ejvs.2013.09.025

[19] Andraska EA, Tran LM, Haga LM et al (2022) Contemporary management of acute and chronic mesenteric ischemia: 10-year experience from a multihospital healthcare system. J Vasc Surg 75:1624–1633.e8 34788652 10.1016/j.jvs.2021.11.040 PMC9038632

[20] Ryer EJ, Kalra M, Oderich GS et al (2012) Revascularization for acute mesenteric ischemia. J Vasc Surg 55:1682–1689 22503176 10.1016/j.jvs.2011.12.017

[21] Davenport DL, Shivazad A, Endean ED (2012) Short-term outcomes for open revascularization of chronic mesenteric ischemia. Ann Vasc Surg 26:447–453 22284770 10.1016/j.avsg.2011.11.006

[22] Tamme K, Reintam Blaser A, Laisaar KT et al (2022) Incidence and outcomes of acute mesenteric ischaemia: a systematic review and meta-analysis. BMJ Open 12:e062846 36283747 10.1136/bmjopen-2022-062846 PMC9608543

[23] Altintas Ü, Lawaetz M, de la Motte L et al (2021) Endovascular treatment of chronic and acute on chronic mesenteric ischaemia: results from a National Cohort of 245 Cases. Eur J Vasc Endovasc Surg 61:603–611 33589326 10.1016/j.ejvs.2021.01.003

[24] Kärkkäinen JM, Lehtimäki TT, Saari P et al (2015) Endovascular therapy as a primary revascularization modality in acute mesenteric ischemia. Cardiovasc Interv Radio 38:1119–1129 10.1007/s00270-015-1064-9 25737456

[25] Omran S, Konietschke F, Mueller V, de Bucourt M, Frese JP, Greiner A (2022) Development of a novel scoring model to estimate the severity grade of mesenteric artery stenosis. J Clin Med 11:7420 10.3390/jcm11247420 PMC9785168 36556035

[26] Nachmias-Peiser N, Soffer S, Horesh N, Zlotnick G, Amitai MM, Klang E (2022) Mortality in patients who underwent computed tomography angiography for a suspected acute mesenteric ischemia as a final alternative diagnosis. Isr Med Assoc J 25:828–833 36573778

[27] Tallarita T, Oderich GS, Gloviczki P et al (2013) Patient survival after open and endovascular mesenteric revascularization for chronic mesenteric ischemia. J Vasc Surg 57:747–755 23332245 10.1016/j.jvs.2012.09.047 PMC3923307

[28] Lima FV, Kolte D, Louis DW et al (2019) Thirty-day readmission after endovascular or surgical revascularization for chronic mesenteric ischemia: Insights from the Nationwide Readmissions Database. Vasc Med 24:216–223 30739588 10.1177/1358863X18816816

[29] Canfora A, Ferronetti A, Marte G et al (2019) Predictive factors of intestinal necrosis in acute mesenteric ischemia. Open Med 14:883–889 10.1515/med-2019-0104 PMC6947755 31934635

[30] Schoots IG, Koffeman GI, Legemate DA, Levi M, van Gulik TM (2004) Systematic review of survival after acute mesenteric ischaemia according to disease aetiology. Br J Surg 91:17–27 14716789 10.1002/bjs.4459

[31] Tran LM, Andraska E, Haga L, Sridharan N, Chaer RA, Eslami MH (2022) Hospital-based delays to revascularization increase risk of postoperative mortality and short bowel syndrome in acute mesenteric ischemia. J Vasc Surg 75:1323–1333.e3 34634418 10.1016/j.jvs.2021.09.033 PMC8991435

[32] Lemma A, Tolonen M, Vikatmaa P et al (2022) Epidemiology, diagnostics, and outcomes of acute occlusive arterial mesenteric ischaemia: a population based study. Eur J Vasc Endovasc Surg 64:646–653 10.1016/j.ejvs.2022.07.006 35931276

[33] Nogueira RG, Jadhav AP, Haussen DC et al (2018) Thrombectomy 6 to 24 h after stroke with a mismatch between deficit and infarct. N Engl J Med 378:11–21 29129157 10.1056/NEJMoa1706442

[34] von Erffa J, Ropers D, Pflederer T et al (2008) Differentiation of total occlusion and high-grade stenosis in coronary CT angiography. Eur Radiol 18:2770–2775 18604538 10.1007/s00330-008-1068-9

[35] Copin P, Ronot M, Nuzzo A et al (2018) Inter-reader agreement of CT features of acute mesenteric ischemia. Eur J Radiol 105:87–95 30017304 10.1016/j.ejrad.2018.05.027

