# Supplementary material for: Acute arterial mesenteric ischaemia: comparison of partial and complete occlusion of the superior mesenteric artery
Source: Insights Imaging. 2025 May 8;16:97. doi: 10.1186/s13244-025-01986-8 (PMC12062469; doi:10.1186/s13244-025-01986-8)
Supplement: Supplementary file 1 — ELECTRONIC SUPPLEMENTARY MATERIAL [file 13244_2025_1986_MOESM1_ESM.pdf]

Acute arterial mesenteric ischaemia: comparison of partial and complete occlusion of the superior mesenteric artery

ELECTRONIC SUPPLEMENTARY MATERIAL

Treatment strategy and management algorithm.

After admission, all patients received the multimodal medical protocol, including oral antibiotics (12), anti-platelet and anticoagulation therapy, bowel rest, and blood volume resuscitation.

When technically possible, endovascular revascularization was proposed as the first line treatment in patients with a low probability of extensive intestinal necrosis (i.e., Clichy score of 0-1). The Clichy score weights the probability of transmural necrosis based on the serum lactate value, the presence of organ failure(s) and small bowel dilatation on CT (8). If endovascular treatment failed, did not improve, or worsened the clinical status, laboratory tests or follow-up imaging, patients were referred for surgical bowel exploration and/or open revascularization, when needed. Laparoscopy was performed to evaluate the presence of bowel necrosis in patients with an initially high probability of intestinal necrosis (e.g., Clichy score of 2-3, bowel pneumatosis, organ failure, or peritonitis). When intestinal necrosis was suggested visually (no fluorescence tool was used), resection was undertaken, and an enterostomy and/or colostomy was created. Bowel continuity was restored, if possible, four to six weeks after intestinal resection.

Endovascular revascularization methods included stenting, thrombus-aspiration, and in-situ thrombolysis and the choice of method(s) was usually decided on pre-treatment CT depending on the aetiology and lesion localization. Transluminal angioplasty with stenting was typically the first line treatment for proximal lesions

(segment S1, embolic or atherothrombotic). Thrombus-aspiration was favored for proximal (S1) and middle (S2) lesions. Isolated catheter-directed intra-arterial thrombolysis was attempted in case of distal clots (i.e., S3 not accessible to thrombus-aspiration). Stenting used covered stents (BeGraft, Bentley InnoMed GmbH, Hechingen, Germany). Thrombus-aspiration was performed manually (using a Mach 1™ catheter, Boston Scientific, Marlborough, MA, USA) with a 50-mL syringe or mechanically (Penumbra aspiration pump with a specific catheter; Indigo Catheter, Penumbra, Inc., Alameda, CA, USA). Thrombus aspiration was mostly performed for clots located in the middle portion of the SMA (S2), and attempts were limited to three passes. When complete recanalization of all branches could not be obtained, catheter-directed thrombolysis (using Fountain Infusion System [Merit Medical, South Jordan, UT, USA] with a bolus of 8 mg of Alteplase [Actilyse, Boehringer Ingelheim, Ingelheim am Rhein, Germany], or 100,000 IU of urokinase [Actosolv, Eumedica, Manage, Belgium] followed by 1mg/h of alteplase or 100,000 UI/hour delivered with a syringe pump) could be used in addition to thrombus-aspiration. Open revascularization included thrombectomy with a Fogarty catheter, retrograde open mesenteric stenting (arteriotomy is performed after laparotomy and then stenting is executed under fluoroscopic guidance), or mesenteric bypass. Open techniques were performed when endovascular techniques were not feasible or failed. The delay between the initial CT and revascularization, as well as the type of upfront revascularization (endovascular or open) were recorded. Reperfusion injury was also noted (13).

Outcomes were recorded, in particular the need for surgical resection and the rate of transmural necrosis on pathology as well as any features of gastrointestinal disability (i.e., short bowel syndrome, persistent parenteral nutritional support, definite stoma).

Supplemental Figures

Figure 1 Abdominal CT-scan of a 82 years old patient with AMI showing an complete occlusion of the S2 segment of the SMA (arrow) : (A) axial view and (B) coronal view

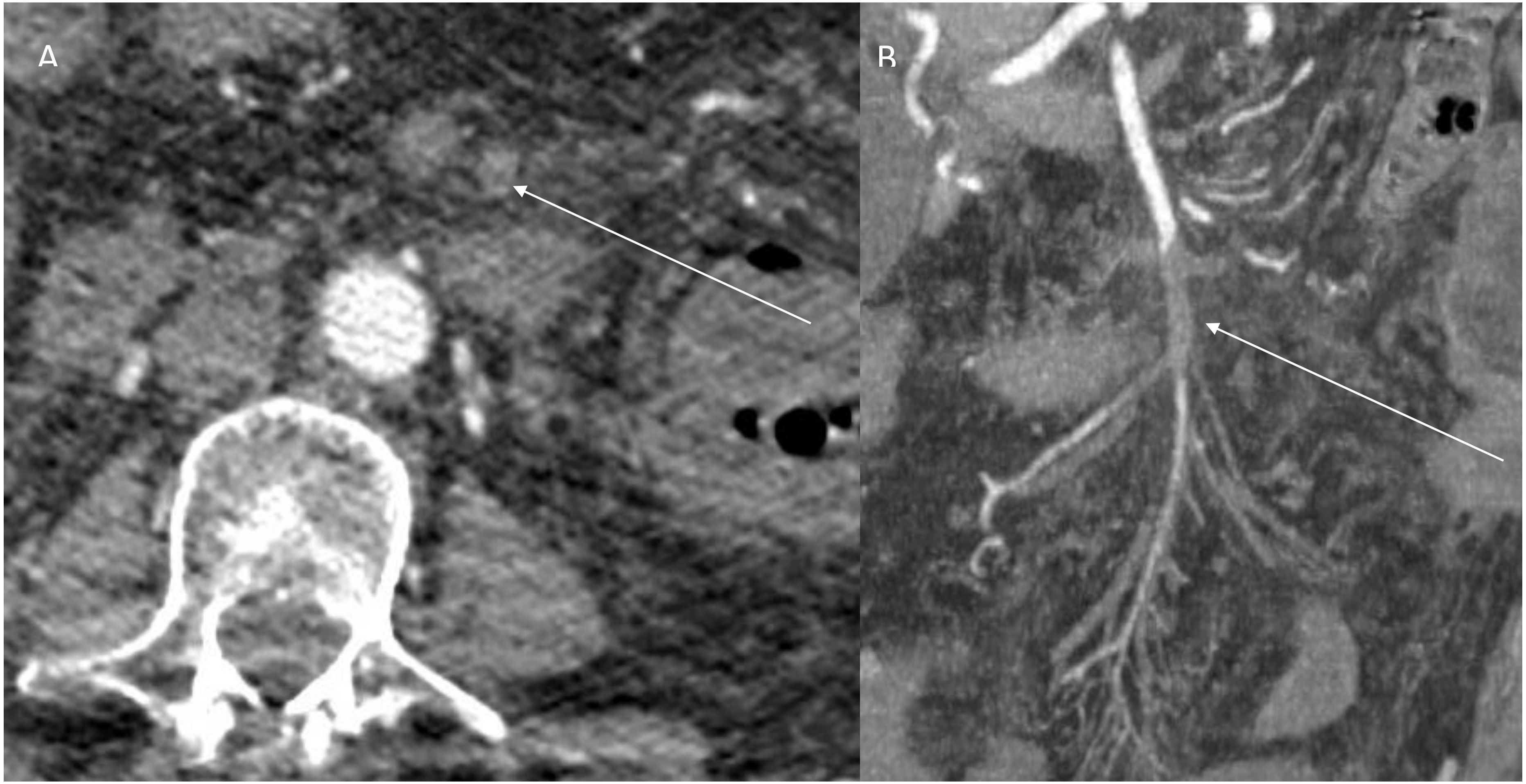

Figure 2 Abdominal CT-scan of a 80 years old patient with AMI showing an complete occlusion of the S2 and S3 segment of the SMA (arrow) : (A) axial view and (B) coronal view

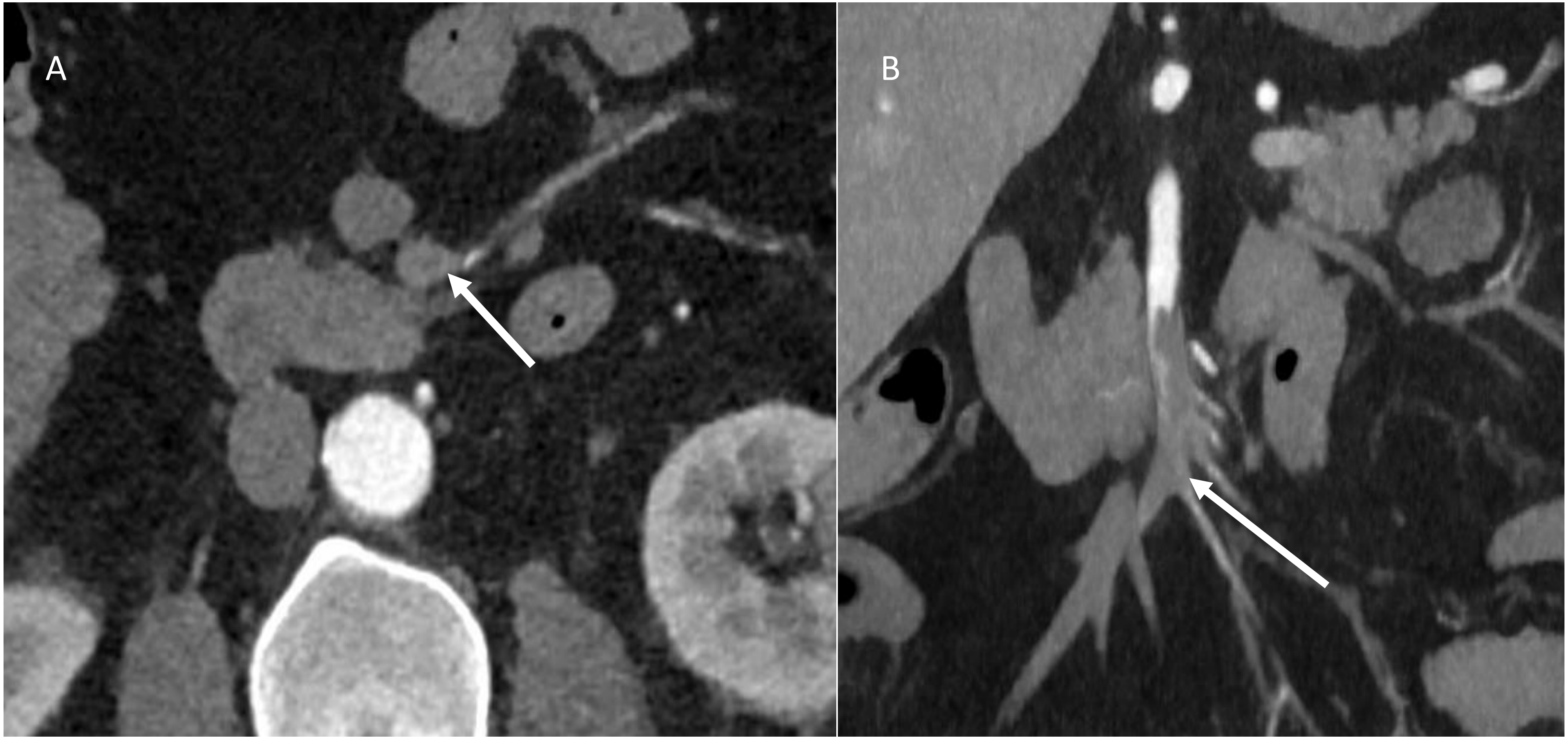

Figure 3 Abdominal CT-scan of a 79 years old patient with AMI showing an complete occlusion of the S0 segment of the SMA (arrow) : (A) axial view and (B) sagittal view

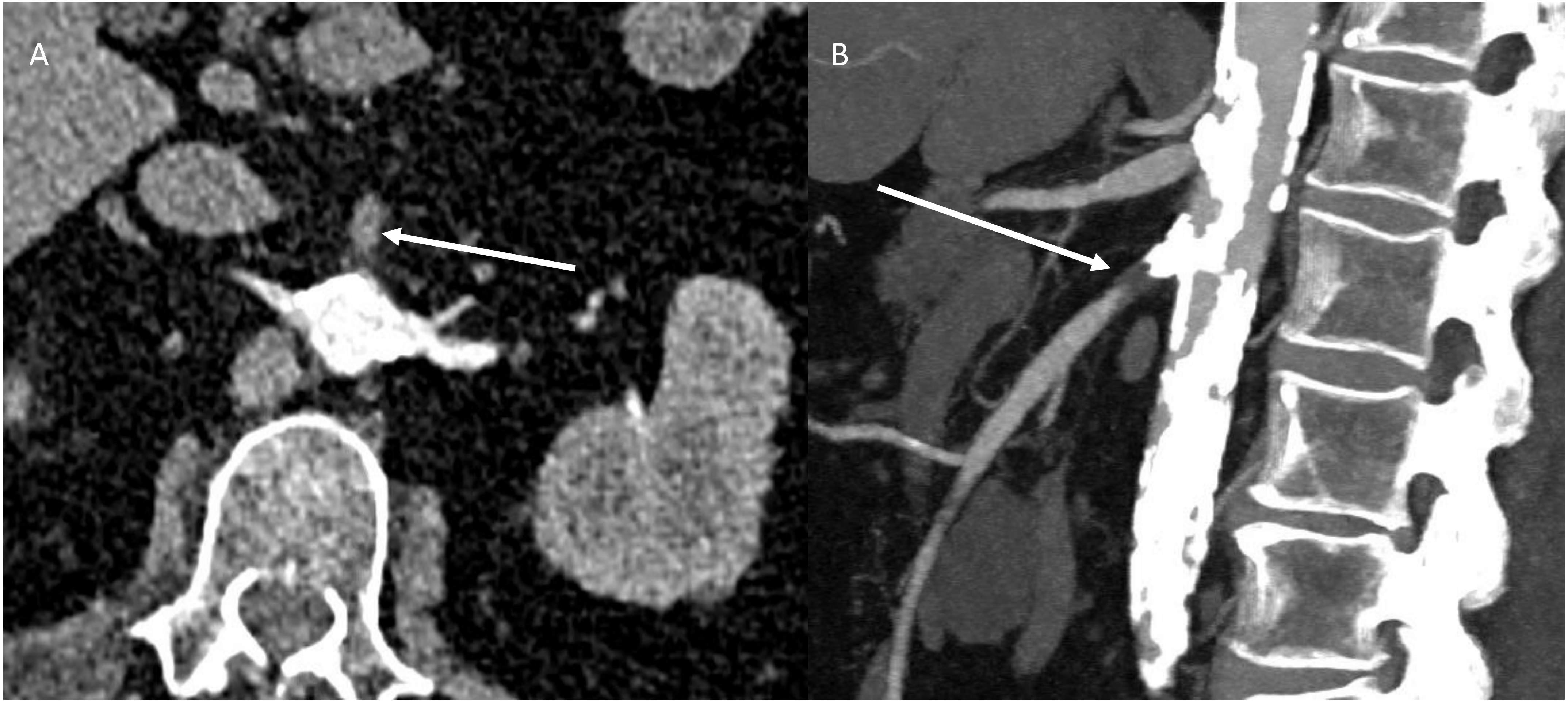

Figure 4 Abdominal CT-scan of a 77 years old patient with AMI showing an incomplete occlusion of the S2 segment of the SMA (arrow) : (A) axial view and (B) sagittal view

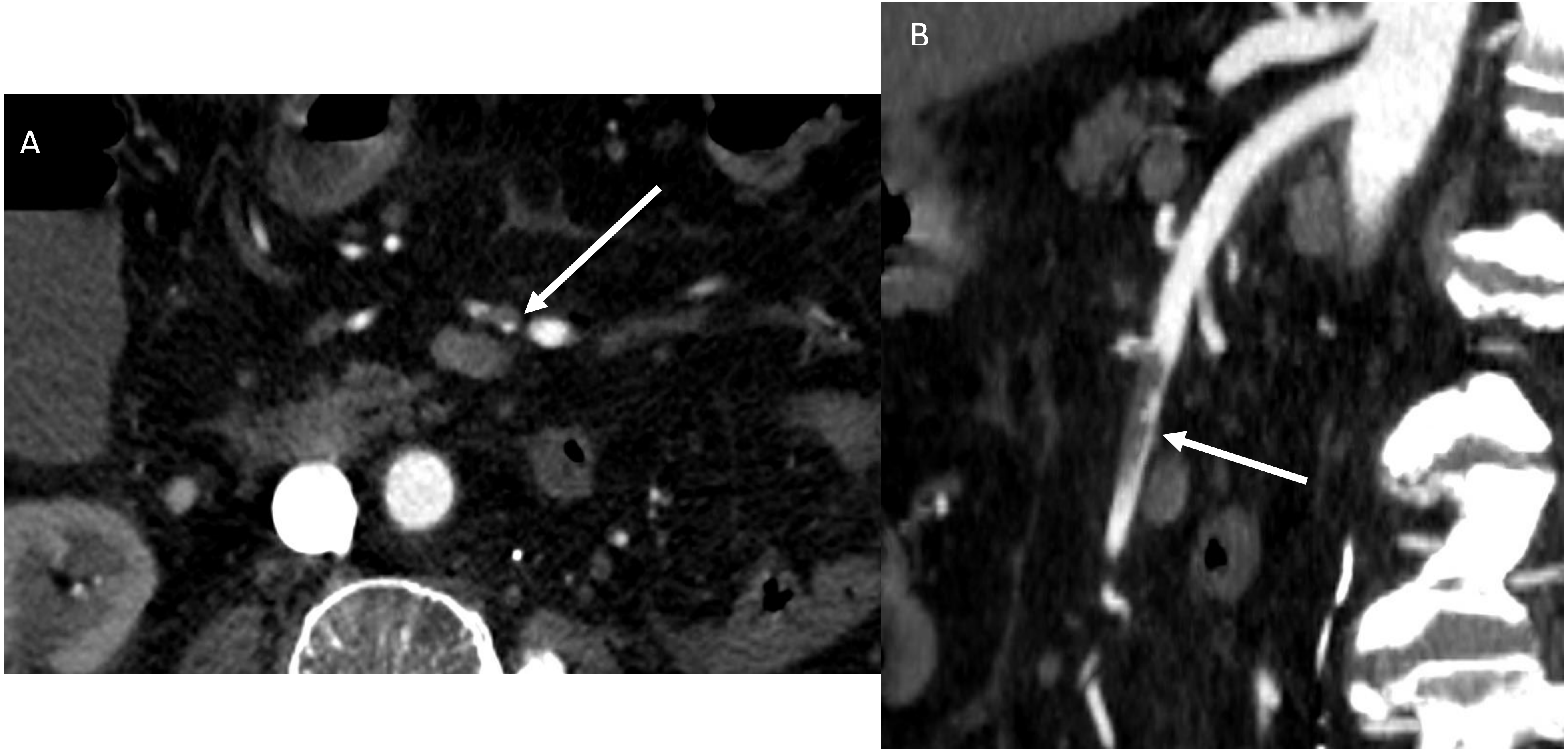

Figure 5 Abdominal CT-scan of a 45 years old patient with AMI showing an incomplete occlusion of the S1 segment of the SMA (arrow) : (A) coronal view and (B) sagittal view

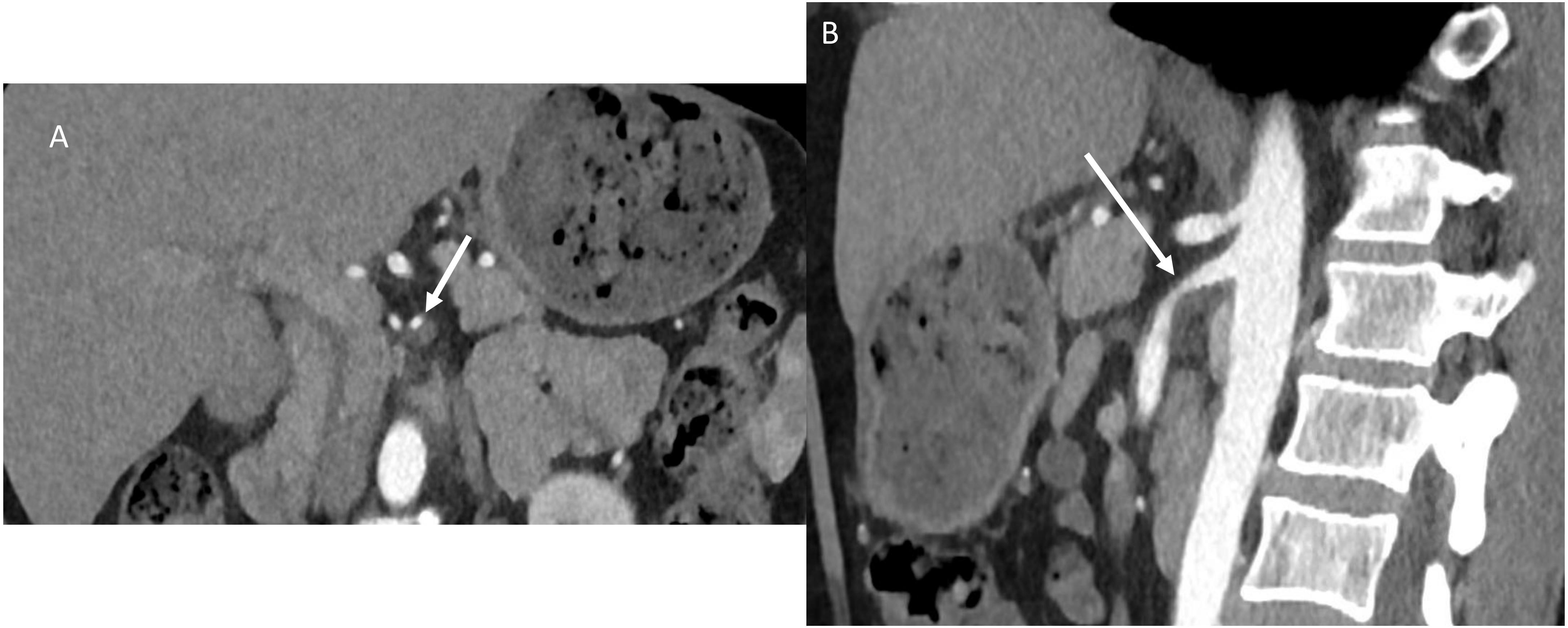

Figure 6 Abdominal CT-scan of a 87 years old patient with AMI showing an incomplete occlusion of the S2 segment of the SMA (arrow) : (A) axial view and (B) coronal view

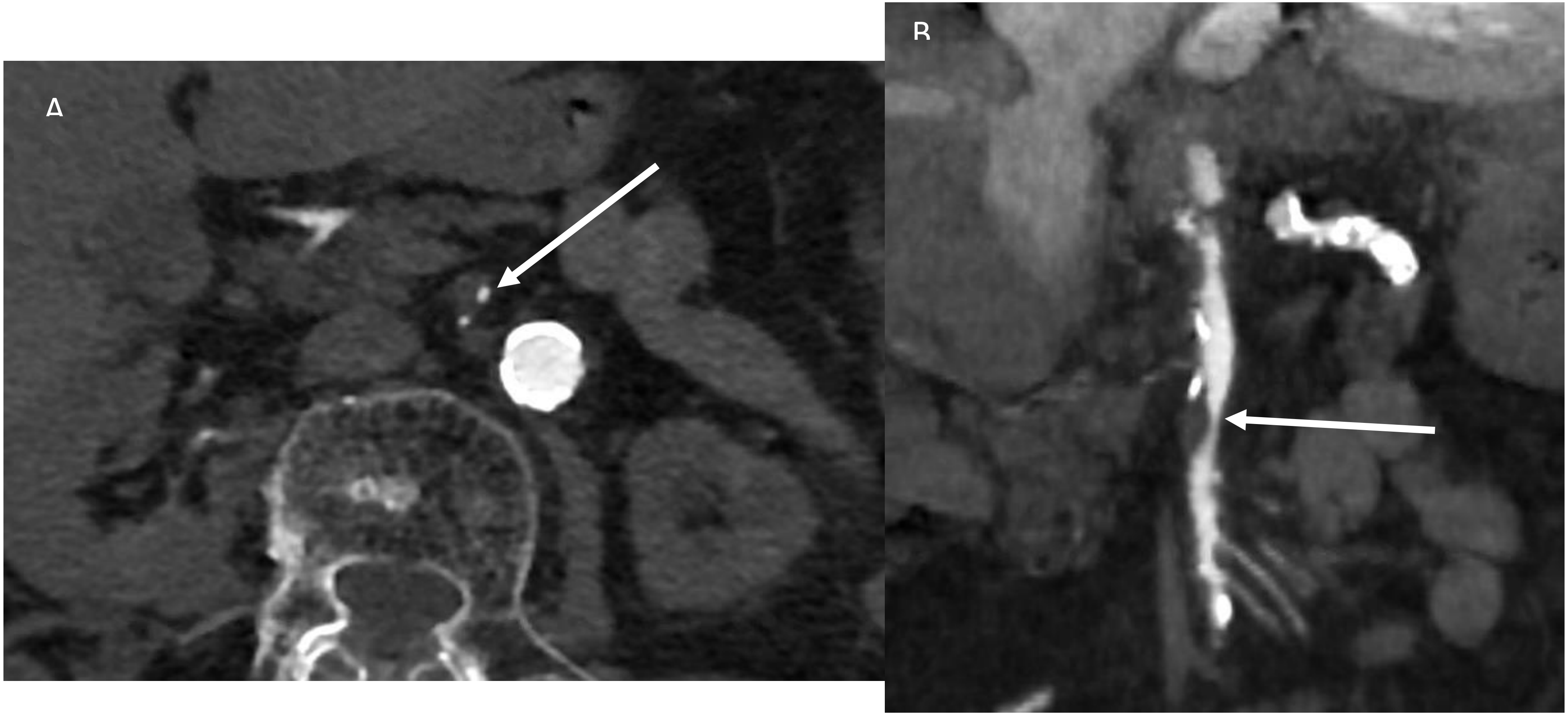

Figure 7 Abdominal CT-scan of a 56 years old patient with AMI showing an incomplete occlusion of the S0 segment of the SMA (arrow) : (A) coronal view and (B) sagittal view

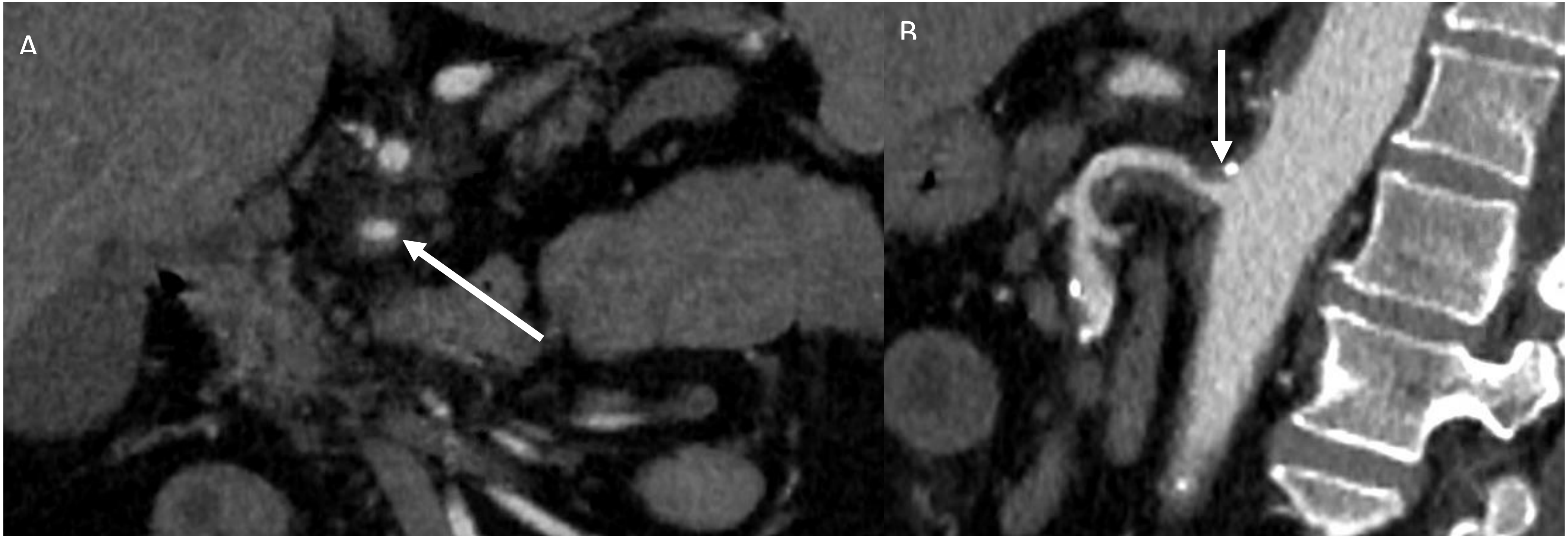

Supplementary Table 1 CT parameters for CTs performed in our centre

	**Non contrast phase**	**Arterial phase**	**Portal-venous phase**
Position	Supine	Supine	Supine
Coverage	Liver dome to pubic symphysis	Liver dome to pubic symphysis	Liver dome to pubic symphysis
Peak beam ernergy	120 kv	120 kv	120kv
mA mode	Modulated	Modulated	Modulated
Pitch	1.375	0.984	0.984
Slice Thickness	2.5 mm	1.25 mm	1.25 mm
Image reconstruction overlap	1.25 x 1 mm	1.25 x 1 mm	1.25 x 1 mm
Field of view	Large	Large	Large
Acquisition time		Bolus-triggered (beginning of the abdominal aorta) at 120 UH threshold	50 sec after arterial phase
Iodine concentration		350 mg/mL	
Contrast media nature		iobitridol, iomeprol	
Contrast media dose		2 mL/kg	
Contrast media Flow rate		4 mL/sec	
